# Nasal Cytology as a Cellular Window into Epithelial Dysfunction and Type 2 Inflammation: From Mechanisms to Translational Implications

**DOI:** 10.3390/cells15040323

**Published:** 2026-02-10

**Authors:** Matteo Gelardi

**Affiliations:** Unit of Otolaryngology, Department of Clinical and Experimental Medicine, University of Foggia, 71122 Foggia, Italy; matteo.gelardi@unifg.it

**Keywords:** nasal cytology, epithelial barrier dysfunction, type 2 inflammation, chronic rhinosinusitis with nasal polyps, cellular phenotypes, precision medicine

## Abstract

Epithelial barrier dysfunction is increasingly recognized as a central pathogenic mechanism in chronic inflammatory airway diseases characterized by type 2 immune responses. Chronic rhinosinusitis with nasal polyps (CRSwNP) represents a paradigmatic condition in which structural epithelial alterations, impaired barrier integrity, and sustained release of epithelial-derived alarmins interact with innate and adaptive immune pathways to drive persistent inflammation and tissue remodeling. In this context, understanding disease heterogeneity requires tools capable of capturing cellular and immunological complexity beyond purely molecular or symptom-based classifications. Nasal cytology is a standardized, minimally invasive, and repeatable technique that provides direct in vivo assessment of epithelial morphology and inflammatory cell infiltrates at the mucosal surface. By identifying distinct cytological patterns, including eosinophil-dominant, mast cell-rich, and mixed inflammatory signatures, nasal cytology reflects the underlying immunopathological mechanisms of CRSwNP and correlates with disease severity, clinical control, and therapeutic responsiveness. Its dynamic nature allows longitudinal monitoring of inflammatory changes over time, offering insights that complement clinical evaluation and endoscopic assessment. This review integrates current knowledge on epithelial barrier dysfunction and type 2 inflammation with the translational relevance of nasal cytology in CRSwNP. Particular attention is given to its role in disease phenotyping, prognostic stratification, and monitoring of biologic therapies. Within precision medicine frameworks, nasal cytology emerges as a robust cellular biomarker bridging epithelial biology, immune profiling, and personalized clinical decision-making.

## 1. Introduction

Chronic rhinosinusitis with nasal polyps (CRSwNP) is a complex and heterogeneous inflammatory disorder characterized by persistent sinonasal symptoms, marked impairment of quality of life, and a high rate of recurrence despite optimal medical and surgical management. Over the past two decades, CRSwNP has progressively shifted from being considered a purely anatomical or infective condition to a paradigmatic example of chronic inflammatory disease driven by distinct immunological endotypes, with type 2 inflammation representing the most prevalent and clinically relevant pattern in Western populations [1,2]. Although eosinophilic inflammation represents the predominant endotype of CRSwNP in Western populations, a clinically relevant proportion of patients exhibits non-eosinophilic or mixed inflammatory patterns, including neutrophilc and eosinophil-mast cell phenotypes, in which nasal cytology may provide additional diagnostic and prognostic value [3,4].

At the center of this paradigm shift lies the concept of epithelial barrier dysfunction. The sinonasal epithelium is no longer viewed as a passive physical boundary but rather as an active immunological interface capable of sensing environmental stimuli, orchestrating immune responses, and shaping chronic inflammation. Structural alterations of tight junctions, impaired epithelial integrity, abnormal epithelial remodeling, and dysregulated release of epithelial-derived alarmins such as thymic stromal lymphopoietin, interleukin-25, and interleukin-33 collectively contribute to the initiation and maintenance of type 2–skewed immune responses. These mechanisms foster eosinophilic inflammation, mast cell activation, and persistent tissue remodeling, ultimately leading to polyp formation and disease chronicity [5,6].

While molecular and histopathological approaches have greatly expanded our understanding of these processes, they often remain confined to ex vivo analyses and lack immediate clinical applicability in daily practice. In this context, there is a growing need for translational tools capable of bridging epithelial biology, immune mechanisms, and clinical phenotypes in a reproducible and patient-centered manner.

Rather than providing a comprehensive overview of CRSwNP pathophysiology, the present review deliberately focuses on epithelial barrier dysfunction and type 2 inflammation through the lens of nasal cytology. Nasal cytology represents a unique in vivo window into the cellular microenvironment of the sinonasal mucosa, allowing direct observation of epithelial cells and inflammatory infiltrates under real-life conditions. Unlike histology, which captures a static and localized snapshot of tissue architecture, nasal cytology offers a dynamic and repeatable assessment of disease activity, reflecting ongoing epithelial–immune interactions over time [7,8].

By identifying distinct cytotypes and cellular patterns, including eosinophils, mast cells, neutrophils, and their combinations, nasal cytology provides clinically meaningful insights into disease severity, inflammatory burden, and therapeutic responsiveness. Importantly, it enables the recognition of complex inflammatory phenotypes that cannot be adequately captured by single biomarkers or by eosinophil counts alone. In this sense, nasal cytology functions not merely as a diagnostic technique, but as a translational tool capable of integrating epithelial dysfunction, immune activation, and clinical expression of CRSwNP [9].

The aim of this review is therefore to integrate current knowledge on epithelial barrier alterations and type 2 inflammation with the clinical and translational relevance of nasal cytology in CRSwNP. By framing nasal cytology as a bridge between basic epithelial biology and precision medicine, this review seeks to highlight its role in disease stratification, prognostic assessment, and personalized therapeutic decision-making in modern rhinology.

This narrative review focuses on nasal cytology as a clinical and translational tool to investigate epithelial barrier dysfunction and type 2 inflammation in chronic rhinosinusitis with nasal polyps. To preserve a clear and methodologically coherent scope, therapeutic approaches or adjunctive treatments not directly related to cytological or epithelial mechanisms were intentionally excluded.

## 2. Methods

This article was conceived as a narrative review aimed at exploring nasal cytology as a translational tool linking epithelial dysfunction with type 2 inflammation in chronic rhinosinusi2is with nasal polyps.

The review was conducted in accordance with the SANRA (Scale for the Assessment of Narrative Review Articles) recommendations [10] A literature search was performed using PubMed and Scopus databases, including articles published in English up to January 2026. The search strategy was based on combinations of keywords such as nasal cytology, chronic rhinosinusitis with nasal polyps, type 2 inflammation, eosinophils, mast cells, neutrophils, epithelial barrier dysfunction, tissue remodeling, and biologic therapies.

Articles were selected according to their relevance to the pathophysiological mechanisms, clinical applicability, and translational implications of nasal cytology in inflammatory airway diseases. Given the narrative nature of the review, the selection and interpretation of studies followed a hypothesis-driven and interpretative approach rather than a systematic quantitative synthesis [11].

Self-citations were included when original methodological contributions, longitudinal clinical observations, or conceptual frameworks developed by the author were directly relevant to the topics discussed. This approach reflects the highly specialized nature of the field and the limited number of research groups involved in the standardization and clinical application of nasal cytology.

## 3. Epithelial Barrier Dysfunction in CRSwNP

The nasal epithelium constitutes the first physical and immunological barrier between the external environment and the underlying immune system. Under physiological conditions, this highly specialized structure ensures mucosal protection through tightly regulated intercellular junctions, mucociliary clearance, and controlled immune surveillance. In chronic rhinosinusitis with nasal polyps, however, this barrier function is profoundly disrupted, representing a key initiating and perpetuating factor of chronic inflammation [12,13].

Multiple studies have demonstrated that epithelial integrity in CRSwNP is compromised at both structural and functional levels. Alterations in tight junction proteins such as occludin, claudins, and zonula occludens lead to increased epithelial permeability, facilitating the penetration of allergens, pathogens, and irritants into the subepithelial compartment. This enhanced permeability not only exposes immune cells to persistent environmental stimuli but also sustains a state of chronic epithelial stress and injury. In parallel, epithelial remodeling processes, including basal cell hyperplasia, goblet cell metaplasia, and impaired epithelial regeneration, further weaken barrier resilience and contribute to disease chronicity [12,13].

Beyond its mechanical role, the damaged epithelium actively participates in immune orchestration. Epithelial injury and stress trigger the release of alarmins, notably thymic stromal lymphopoietin, interleukin-25, and interleukin-33. These cytokines act as upstream regulators of type 2 inflammation by activating innate lymphoid cells type 2, dendritic cells, mast cells, and Th2 lymphocytes. The result is an amplified inflammatory cascade characterized by eosinophil recruitment, mast cell activation, local IgE production, and persistent tissue remodeling [2,6,12].

Importantly, epithelial-derived alarmins represent a biological bridge between barrier dysfunction and immune polarization. Their sustained release creates a self-perpetuating loop in which epithelial damage fuels inflammation, and inflammation, in turn, further compromises epithelial integrity. This vicious cycle provides a compelling explanation for the chronicity and high recurrence rate of CRSwNP, even after surgical intervention [1,12].

From a translational perspective, the recognition of epithelial barrier dysfunction as a central pathogenic mechanism has profound therapeutic implications. It provides the biological rationale for targeting upstream inflammatory pathways rather than downstream effector cells alone. Therapeutic strategies aimed at modulating epithelial-derived cytokines or restoring barrier function have therefore emerged as promising approaches in the management of severe and recurrent CRSwNP [14,15].

Within this framework, the assessment of epithelial damage and its inflammatory consequences in vivo becomes crucial. While molecular and histological analyses offer valuable mechanistic insights, they are limited by invasiveness and lack of repeatability. This gap underscores the need for complementary tools capable of capturing epithelial–immune interactions dynamically at the mucosal surface, setting the stage for the role of nasal cytology in translating epithelial barrier dysfunction into clinically actionable information [8,16].

## 4. Type 2 Inflammation and Cellular Signatures

Type 2 inflammation represents the dominant immunological paradigm in chronic rhinosinusitis with nasal polyps, particularly in Western populations [2]. It is classically characterized by eosinophilic infiltration, mast cell activation, and a cytokine milieu dominated by interleukin-4, interleukin-5, and interleukin-13. These mediators orchestrate IgE production, tissue eosinophilia, mucus hypersecretion, and progressive remodeling of the sinonasal mucosa, ultimately sustaining polyp growth and disease persistence [1,3].

However, growing evidence indicates that type 2 inflammation in CRSwNP cannot be adequately explained by eosinophilia alone [3,17]. Mast cells have emerged as critical amplifiers of inflammation, interacting closely with eosinophils, epithelial cells, and fibroblasts within the polyp microenvironment [2,17]. Through the release of proteases, lipid mediators, cytokines, and growth factors, mast cells contribute to epithelial damage, vascular permeability, and fibrotic remodeling, reinforcing the chronic inflammatory state. The coexistence and spatial proximity of eosinophils and mast cells appear to define a more severe and recalcitrant inflammatory phenotype, challenging traditional mono-parametric classifications [17].

At the tissue level, type 2 inflammation reflects a complex network of interactions involving epithelial cells, stromal elements, and immune populations. Fibroblasts, once considered passive structural components, actively participate in inflammatory signaling and extracellular matrix remodeling, while epithelial cells act as both sensors and effectors of immune responses. This dynamic crosstalk shapes the cellular architecture of nasal polyps and contributes to their biological heterogeneity [2,13].

In addition to epithelial and immune cells, increasing evidence has identified mesenchymal stem/stromal cells (MSCs) as an integral component of the nasal polyp microenvironment [18,19]. MSCs have been isolated from both common nasal polyps and antrochoanal polyps, where they display multipotent differentiation capacity and marked immunomodulatory activity [20]. Experimental studies have demonstrated that polyp-derived MSCs are able to interact with T lymphocytes, modulate cytokine expression, and influence local inflammatory polarization, thereby contributing to the persistence of type 2 immune activation [21].

Alterations in the functional properties of MSCs derived from nasal polyps, including impaired adipogenic differentiation and dysregulated immunoregulatory signaling, further suggest a role for these cells in tissue remodeling and inflammation-associated epithelial plasticity. Although mesenchymal stem cells are not directly detectable by nasal cytology, their biological activity provides an important stromal context that helps explain the complexity and chronicity of inflammatory patterns observed at the epithelial surface. In this framework, MSCs can be viewed as upstream modulators that indirectly shape the cytological phenotypes identified in vivo, reinforcing the concept of CRSwNP as a multicellular and highly integrated inflammatory disease.

Recent advances in single-cell transcriptomics have further refined this concept, revealing profound epithelial and stromal cell heterogeneity within nasal polyp tissue [13]. These studies have identified distinct cellular subsets characterized by specific transcriptional programs associated with disease severity, persistence, and therapeutic resistance. Alterations in genes involved in barrier integrity, cytokine signaling, and tissue remodeling underscore the central role of cellular diversity in driving heterogeneous clinical outcomes. Importantly, these findings reinforce the notion that CRSwNP is not a single disease entity but rather a spectrum of related inflammatory conditions sharing common clinical features [2].

Chronic epithelial injury and sustained exposure to inflammatory mediators may also promote epithelial–mesenchymal transition (EMT), a process by which epithelial cells progressively lose polarity and junctional organization while acquiring mesenchymal-like features. In CRSwNP, EMT has been linked to prolonged type 2 inflammation, epithelial stress, and remodeling signals driven by cytokines and growth factors within the inflamed mucosa [22,23].

EMT represents a critical biological bridge between epithelial barrier dysfunction and stromal remodeling, contributing to fibrosis, altered tissue architecture, and impaired epithelial regeneration. This process further amplifies the vicious cycle in which epithelial damage fuels inflammation, and inflammation in turn perpetuates epithelial instability. From a translational perspective, EMT reinforces the concept that epithelial alterations observed at the cytological level are not merely descriptive but mirror deeper structural and functional reprogramming of the sinonasal mucosa.

While high-dimensional molecular techniques have substantially advanced mechanistic understanding, their translation into routine clinical practice remains limited. In contrast, cellular-level assessment at the mucosal surface offers a pragmatic and clinically meaningful approach to capturing inflammatory complexity. By directly identifying the relative contribution and interaction of key effector cells, a cellular perspective allows the stratification of type 2 inflammation beyond simplified endotypes.

In this context, nasal cytology emerges as a valuable translational tool capable of mirroring the cellular signatures of type 2 inflammation in vivo [8,16]. Through the identification of specific cytological patterns, including eosinophil-dominant, mast cell–rich, and mixed eosinophil–mast cell infiltrates, nasal cytology provides an accessible and repeatable method for translating immunopathological complexity into clinically actionable information [4,24]. This cellular approach lays the foundation for integrated grading systems and personalized therapeutic strategies, which will be explored in the following sections.

## 5. Nasal Cytology: Technique and Cytological Patterns

Nasal cytology is a well-established, standardized, and minimally invasive technique that enables direct visualization of epithelial cells and inflammatory infiltrates at the level of the nasal mucosa. Sampling is typically performed by gentle scraping of the inferior turbinate, allowing the collection of superficial epithelial layers without causing patient discomfort or tissue damage. The procedure is rapid, repeatable, and suitable for routine clinical practice, making it particularly valuable for both diagnostic evaluation and longitudinal disease monitoring [25,26].

**Figure 1:**
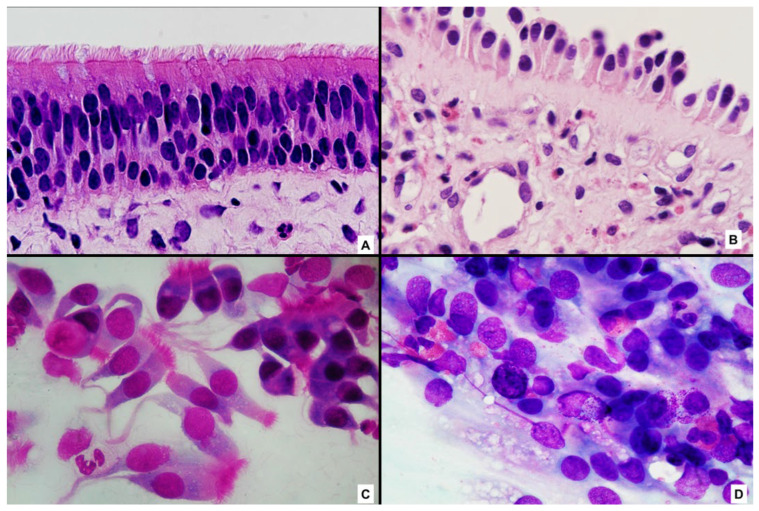
Epithelial barrier integrity and cytological correlates in healthy nasal mucosa and nasal polyposis. (**A**) Histology of healthy nasal mucosa showing a well-organized pseudostratified ciliated epithelium with preserved epithelial integrity. (**B**) Histology of nasal polyposis showing de-epithelialization, basement membrane thickening, and prominent eosinophilic inflammatory infiltration. Hematoxylin and eosin staining, original magnification ×400. (**C**) Normal nasal cytology characterized by numerous well-preserved ciliated epithelial cells, indicative of epithelial homeostasis. (**D**) Nasal cytology from nasal polyposis showing a dense type 2 inflammatory infiltrate with numerous eosinophils and mast cells, some displaying signs of degranulation. May–Grünwald–Giemsa staining, original magnification ×1000. All images were generated in the author’s laboratory. The histological and cytological images are representative examples included for didactic and conceptual purposes, to illustrate epithelial barrier integrity and its cytological correlates discussed in the text, rather than to present new experimental data.

In physiological conditions, nasal cytology reveals a predominance of well-preserved ciliated epithelial cells with intact morphology, reflecting normal epithelial turnover and barrier integrity. In contrast, patients with chronic rhinosinusitis with nasal polyps exhibit marked cytological alterations, including epithelial cell damage, loss of ciliation, cytoplasmic vacuolization, and increased cellular debris. These epithelial changes are consistently accompanied by inflammatory infiltrates whose composition varies according to the underlying immunopathological mechanisms (Figure 1A–D).

In addition to eosinophilic inflammation, nasal cytology allows the identification of neutrophilic and mixed inflammatory patterns in CRSwNP. Although less prevalent in Western populations, non-type 2 and mixed endotypes account for a clinically relevant proportion of CRSwNP cases and are often associated with disease severity, persistent symptoms, and reduced responsiveness to corticosteroids and biologic therapies [3,14,27].

Neutrophil-predominant cytological patterns have been linked to epithelial barrier dysfunction, recurrent infections, and biofilm-related inflammation, highlighting a distinct pathophysiological trajectory compared with eosinophilic disease [28]. In this context, nasal cytology represents a valuable tool for identifying neutrophilic inflammation at the epithelial level, providing clinically actionable information that cannot be captured by systemic biomarkers alone and supporting a more precise endotyping of CRSwNP. Despite this heterogeneity, eosinophils and mast cells remain the hallmark inflammatory cells observed in CRSwNP, with eosinophil-dominant cytotypes typically associated with type 2-driven inflammation and correlating with increased disease severity, higher recurrence rates, and reduced responsiveness to conventional therapies [9,24]. Mast cells, often overlooked in routine assessments, play a critical role in amplifying inflammation and tissue remodeling. Their presence, either alone or in combination with eosinophils, defines distinct cytological patterns that reflect more aggressive and persistent disease phenotypes [4].

Importantly, mixed eosinophil–mast cell cytotypes represent a particularly severe inflammatory signature, mirroring the complex cellular interactions observed in tissue studies. These patterns provide in vivo confirmation of the synergistic role of eosinophils and mast cells in sustaining epithelial damage, vascular permeability, and chronic inflammation. From a clinical perspective, the identification of such cytotypes allows a more nuanced stratification of patients beyond simplistic binary classifications [4,25].

One of the most distinctive advantages of nasal cytology lies in its dynamic nature. Unlike histological analysis, which provides a static snapshot of tissue architecture at a single time point, nasal cytology enables repeated assessments over time [8,29]. This characteristic allows clinicians to monitor fluctuations in inflammatory cell populations, evaluate disease evolution, and assess response to medical or biological therapies. Changes in cytological patterns often precede clinical improvement or deterioration, highlighting the potential of nasal cytology as an early indicator of treatment effectiveness or impending relapse.

By integrating epithelial morphology with inflammatory cell profiling, nasal cytology offers a comprehensive and clinically actionable view of CRSwNP at the cellular level. Rather than serving as a mere descriptive technique, it functions as a translational tool capable of linking epithelial barrier dysfunction, immune activation, and clinical expression of disease. These features lay the foundation for structured interpretative models and integrated grading systems, which translate cytological findings into prognostic and therapeutic guidance, as discussed in the following section.

## 6. Clinical Integration of Nasal Cytology: A Practical Algorithm

From a clinical perspective, nasal cytology can be integrated into a practical and reproducible algorithm for the diagnosis, stratification, and longitudinal monitoring of patients with chronic rhinosinusitis with nasal polyps.

During medical treatment, repeated cytological assessments enable dynamic monitoring of inflammatory changes over time. Shifts in cellular patterns, such as a reduction in eosinophilic infiltration or persistence of mast cell or neutrophilic components, may precede clinical improvement or therapeutic failure, offering an early indicator of treatment response.

In patients with severe or recurrent disease, nasal cytology may assist in therapeutic decision-making by identifying cytological profiles associated with poor response to conventional therapy. In this setting, cytology-informed stratification can support the selection and monitoring of advanced treatments, including biologic therapies, and guide follow-up intensity.

**Figure 2:**
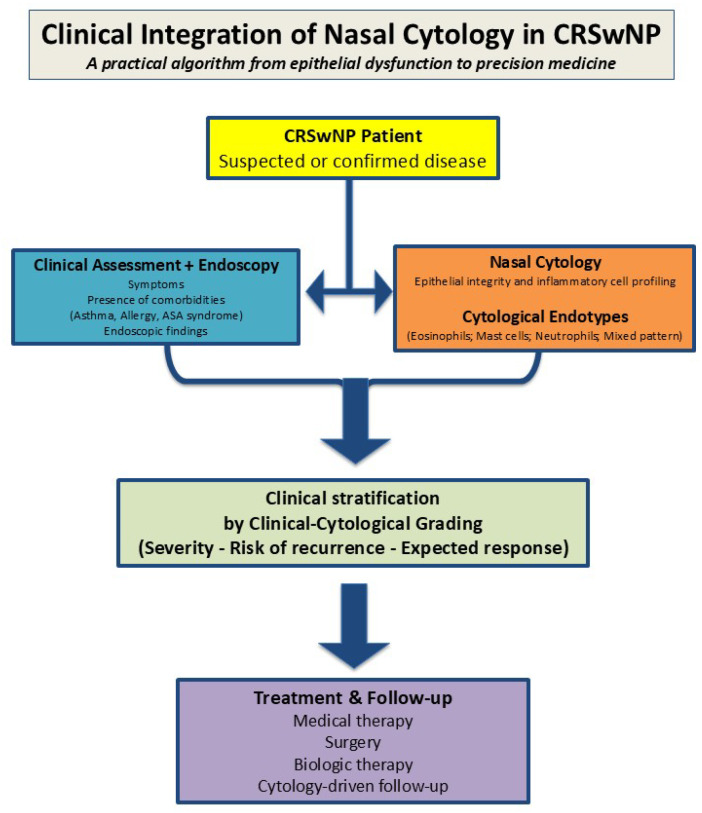
Clinical integration of nasal cytology in CRSwNP. Conceptual schematic representation of a practical clinical algorithm integrating nasal cytology into routine management of chronic rhinosinusitis with nasal polyps (CRSwNP). The figure was created by the author as a conceptual flowchart based on the synthesis of the evidence discussed in the manuscript. It is intended to illustrate the proposed clinical integration of nasal cytology and does not represent original experimental data.

From a clinical standpoint, the integration of nasal cytology into routine management of CRSwNP can be conceptualized as a practical and reproducible algorithm that complements standard clinical and endoscopic assessment (Figure 2).

At the time of diagnosis, nasal cytology complements clinical and endoscopic evaluation by providing direct information on epithelial integrity and inflammatory cell composition.

At diagnosis, nasal cytology complements clinical and endoscopic assessment by providing direct information on epithelial integrity and inflammatory cell composition, allowing early cytological endotyping. Integration of clinical and cytological data supports patient stratification through clinical–cytological grading, informing disease severity, risk of recurrence, and expected therapeutic response. Repeated cytological assessments during follow-up enable longitudinal monitoring and support therapeutic decision-making, including the use of biologic therapies, within a precision medicine framework.

## 7. Translational Implications and Biologic Therapies

The introduction of biologic therapies targeting type 2 inflammatory pathways has profoundly transformed the management of chronic rhinosinusitis with nasal polyps, particularly in patients with severe, recurrent, and corticosteroid-dependent disease. By selectively modulating upstream cytokines and immune signaling cascades, biologics have shifted the therapeutic focus from symptom control to disease modification [14,30]. However, this therapeutic revolution has also highlighted the need for reliable tools capable of identifying suitable candidates, predicting treatment response, and monitoring disease activity over time.

Beyond their clinical efficacy, biologic therapies targeting type 2 inflammatory pathways exert measurable effects on specific cellular subpopulations within the nasal mucosa, including mast cells and eosinophils, as demonstrated by longitudinal cytological changes following dupilumab therapy [31]. Monoclonal antibodies such as dupilumab and omalizumab, as well as mepolizumab, which selectively targets interleukin-5-mediated eosinophil survival and activation, have been shown to induce a significant reduction in eosinophilic infiltration, modulate mast cell involvement, and partially restore epithelial morphology in responsive patients [25,32]. These cellular changes often parallel, and in some cases, precede, clinical improvement, highlighting the importance of cellular-level monitoring.

Clinical studies have incorporated nasal cytology as a non-invasive tool to longitudinally monitor cellular changes during biologic therapy in CRSwNP. In a prospective study of dupilumab-treated patients, nasal swab cytology demonstrated a significant reduction in eosinophilic inflammation over time and correlated with clinical response, although baseline cytology showed limited ability to clearly discriminate responders from non-responders [29].

In this context, nasal cytology offers a unique opportunity to capture the in vivo cellular impact of biologic therapies over time. By documenting shifts in inflammatory cell composition and epithelial integrity, cytology may contribute to the identification of responders and non-responders, support treatment optimization, and provide early signals of disease modulation at the mucosal surface.

In this evolving therapeutic landscape, nasal cytology reinforces its role as a complementary translational tool in precision biologic therapy, enabling the evaluation and longitudinal monitoring of treatment response in CRSwNP. By providing direct in vivo assessment of epithelial integrity and inflammatory cell populations, nasal cytology captures changes at the cellular level that often parallel, and sometimes precede, clinical improvement. Reductions in eosinophil density, modulation of mast cell involvement, and partial restoration of epithelial morphology have been consistently observed in responders to biologic therapy, reflecting effective control of type 2-driven inflammation [29,31].

Importantly, cytological monitoring offers complementary information beyond traditional outcome measures such as symptom scores, quality-of-life questionnaires, and endoscopic findings [1]. While these clinical parameters remain essential, they may not fully capture the underlying immunopathological dynamics, particularly during the early phases of treatment or in cases of discordant clinical responses [8,25]. In contrast, changes in cytological patterns provide objective insights into inflammatory remodeling and immune modulation, enabling a more refined interpretation of therapeutic effectiveness.

From a translational perspective, the value of nasal cytology lies in its ability to integrate biological mechanisms with clinical decision-making. Distinct cytological patterns, including eosinophil-dominant, mast cell-rich, or mixed eosinophil–mast cell infiltrates, have been associated with different disease trajectories and therapeutic responses [4,24]. This cellular stratification supports a move away from uniform treatment algorithms toward a more individualized approach, in which therapy is guided by the dominant inflammatory signature rather than by clinical severity alone.

Recent guidelines and consensus documents increasingly emphasize the need for integrated biomarkers to guide biologic selection, treatment monitoring, and long-term management of CRSwNP [14,30]. Within a treatable trait framework, nasal cytology enables the identification of cellular-driven traits that may inform biologic selection and longitudinal treatment adaptation [33]. In this context, it complements clinical evaluation, endoscopic assessment, and molecular biomarkers by providing a practical, repeatable, and cost-effective method for capturing disease activity at the mucosal surface. Its dynamic nature makes nasal cytology particularly suited for longitudinal follow-up, allowing early detection of relapse, suboptimal response, or inflammatory shifts that may warrant therapeutic adjustment.

By bridging immunopathology and real-world clinical practice, nasal cytology contributes meaningfully to a precision medicine approach in CRSwNP. Rather than serving as a standalone biomarker, it functions as part of an integrated assessment strategy, translating complex cellular and immunological information into actionable insights for personalized biologic therapy. This translational role reinforces the concept that effective management of CRSwNP requires not only advanced therapeutics but also equally sophisticated tools to guide their optimal use [1,34].

## 8. Future Perspectives

Future developments in the management of chronic rhinosinusitis with nasal polyps are expected to further consolidate the role of nasal cytology as a core translational biomarker. One of the most promising directions involves the integration of digital image analysis and artificial intelligence-based systems to enhance the reproducibility, objectivity, and scalability of cytological assessment [35].

Automated cell recognition, quantitative inflammatory scoring, and whole-slide image analysis have the potential to reduce operator dependency and standardize interpretation across centers, thereby facilitating broader clinical adoption.

The application of artificial intelligence to nasal cytology also opens new opportunities for high-throughput analysis and pattern recognition. Machine learning algorithms may identify subtle cytological signatures and inflammatory trajectories that are not readily apparent to the human observer, enabling earlier detection of disease progression, therapeutic response, or impending relapse [36]. These technologies could transform nasal cytology from a semi-quantitative technique into a fully quantitative and predictive tool, aligned with modern precision medicine frameworks.

Beyond technological innovation, the future relevance of nasal cytology lies in its integration within multimodal assessment strategies. Combining cytological data with molecular biomarkers, imaging findings, endoscopic scores, and clinical outcomes may allow the construction of composite models capable of capturing disease complexity at multiple biological levels, particularly in CRSwNP, where heterogeneity in epithelial dysfunction, immune activation, and tissue remodeling underlies variable clinical courses and therapeutic responses [1].

Importantly, nasal cytology is uniquely positioned to serve as a bridge between high-dimensional molecular research and real-world clinical practice. While omics-based technologies provide invaluable mechanistic insights, their routine implementation remains limited by cost, invasiveness, and accessibility. In contrast, nasal cytology offers a practical, repeatable, and patient-friendly method for longitudinal monitoring, making it ideally suited for incorporation into treatable trait-based management algorithms [33].

In parallel, emerging biologic strategies targeting upstream epithelial alarmins, such as thymic stromal lymphopoietin (TSLP), are expected to further expand the therapeutic landscape of CRSwNP in the near future. The introduction of alarmin-directed therapies may reinforce the relevance of epithelial-focused biomarkers, including nasal cytology, for early disease stratification and treatment monitoring [15].

Future research should therefore focus on validating standardized cytological protocols, defining robust quantitative thresholds, and integrating cytology-driven models into clinical decision-making pathways. Prospective studies evaluating the predictive value of cytological patterns for biologic selection, treatment optimization, and long-term outcomes will be essential to fully establish nasal cytology as a cornerstone biomarker in CRSwNP management.

In this evolving landscape, nasal cytology is poised to move beyond its traditional diagnostic role, emerging as a dynamic platform that links epithelial biology, immune profiling, and personalized therapy. By embracing technological innovation and integrative approaches, nasal cytology may play a central role in shaping the next generation of precision medicine strategies in chronic inflammatory airway diseases [37].

## 9. Conclusions

Chronic rhinosinusitis with nasal polyps is a heterogeneous inflammatory disease in which epithelial barrier dysfunction and type 2 immune activation play a central pathogenic role. Within this complex framework, nasal cytology provides a unique in vivo cellular window into the interactions between epithelial damage, inflammatory cell infiltration, and immune amplification at the mucosal surface.

By enabling direct and repeatable assessment of epithelial integrity and inflammatory cell patterns, nasal cytology bridges mechanistic insights from molecular and histological studies with the practical needs of everyday clinical management. The identification of distinct cytological signatures, including eosinophil-dominant, mast cell-rich, and mixed inflammatory patterns, allows refined disease stratification and offers clinically actionable information for therapeutic monitoring.

In the era of biologic therapies and precision medicine, nasal cytology should be considered not merely a diagnostic technique, but a core component of multidimensional CRSwNP assessment. Its integration with clinical, endoscopic, and molecular data represents a concrete step toward more rational, biologically driven, and patient-centered management of chronic inflammatory airway diseases. Future incorporation of cytology-driven algorithms into clinical pathways may redefine the standard of care in CRSwNP management.

## Data Availability

No new data were created or analyzed in this study.
